# The Role of Adrenomedullin as a Predictive Marker of the Risk of Death and Adverse Clinical Events: A Review of the Literature

**DOI:** 10.3390/jcm13164847

**Published:** 2024-08-16

**Authors:** Matteo Antonio Sacco, Saverio Gualtieri, Fabrizio Cordasco, Alessandro Pasquale Tarallo, Maria Cristina Verrina, Aurora Princi, Andrea Bruni, Eugenio Garofalo, Isabella Aquila

**Affiliations:** 1 Institute of Legal Medicine, Department of Medical and Surgical Sciences, ‘Magna Graecia’ University of Catanzaro, 88100 Catanzaro, Italy; matteoantoniosacco@gmail.com (M.A.S.); saveriogualtieri@icloud.com (S.G.); cordasco@unicz.it (F.C.); drtarallomedlegale@gmail.com (A.P.T.); mariacristina.verrina@gmail.com (M.C.V.); aurora.princi@studenti.unicz.it (A.P.); 2 Intensive Care Unit, Department of Medical and Surgical Sciences, ‘Magna Graecia’ University of Catanzaro, 88100 Catanzaro, Italy; andreabruni@unicz.it (A.B.); eugenio.garofalo@unicz.it (E.G.)

**Keywords:** adrenomedullin, sepsis, biomarker, COVID-19, death

## Abstract

Adrenomedullin (ADM) is a vasodilatory peptide that plays a crucial role in maintaining cardiovascular health through its various biological functions. ADM was discovered in the acidic extract of human pheochromocytoma tissue and has been recognized for its significant effects on the vascular system. The main functions of ADM include vasodilation, controlling blood pressure and maintaining vascular integrity, although its role on cardiovascular health is broader. Research has shown that elevated levels of adrenomedullin have been observed in a large number of severe diseases, with high risk of death. In this work, we examined the role of ADM as a predictive molecule of the risk of mortality and adverse clinical outcome through a narrative review of the scientific literature. The results were divided based on the pathologies and anatomical districts examined. This review demonstrates how ADM shows, in many diseases and different systems, a close correlation with the risk of mortality. These results prove the value of ADM as a prognostic marker in various clinical contexts and diseases, with utility in the stratification of the risk of clinical worsening and/or death and in the evaluation of therapeutic efficacy. The results open new perspectives with respect to the concrete possibility that ADM enters clinical practice as an effective diagnostic and prognostic marker of death as well as a molecular target for therapies aimed at patient survival.

## 1. Introduction

In recent years, the discovery of adrenomedullin (ADM) has opened new avenues in cardiovascular physiopathology. This neurohormonal peptide is characterized by a unique amino acid sequence that is highly conserved across different species and it shares structural similarities with calcitonin and the calcitonin gene-related peptide (CGRP). ADM was isolated for the first time in the 90s. In particular, in 1993, Kitamura et al. reported in their work that they had isolated a new peptide with vasoactive properties in human pheochromocytoma [1]. In fact, since the peptide was very abundant in adrenal tissue, the authors coined the neologism of “adrenomedullin”. This molecule consisted of 52 amino acids and was characterized by a hypotensive activity due to a vasodilatory effect [1,2]. Therefore, already at that time, a possible role of the molecule as a hormone involved in the physiology of the cardiovascular system was hypothesized. In the same years, further investigations were conducted demonstrating the existence of a precursor, known as proadrenomedullin N-terminal 20 peptide (PAMP). From a molecular point of view, ADM is a member of the CT/CGRP superfamily whose gene is located on chromosome 11 [3]. The precursor is known as mid-regional pro-adrenomedullin (MR-proADM) [1,2,3,4,5,6,7,8] and is released at a ratio of 1:1 with ADM [3]. The advantage of using MR-proADM in biochemical investigations is related to greater molecular stability, considering that adrenomedullin has a half-life of only 20 min.

The expression and distribution of adrenomedullin in the human body are extensive, reflecting its significance in various physiological processes. Although the peptide was initially isolated in the adrenal medulla, the literature has actually shown that this molecule is ubiquitous [3,4,5]. Research has shown that the adrenomedullin gene is highly expressed in endothelial cells, even more than that expressed in the adrenal medulla where it was first discovered [1]. This peptide is widely distributed across different tissues, particularly in those belonging to the cardiovascular and endocrine systems, where it plays a critical role in homeostasis and the body’s response to injury or stress. Moreover, ADM and its gene are found in a variety of cells and tissues across the human body, including the following: vascular endothelial cells, smooth muscle cells, and adventitial fibroblasts; mucosal epithelium, glandular duct cells, neuroendocrine cells, and the smooth muscle cells of various organs; key organs such as the brain, pituitary gland, heart, kidneys, and gastrointestinal trac; and immune cells, including macrophages and T-cells, which suggests immunomodulatory properties [2,3,4,5,6,7,8,9,10]. This widespread expression of ADM not only highlights its multifunctional role but also indicates its potential as a therapeutic target in various diseases. Further studies have highlighted its presence in human thymic tissue, suggesting a role in immune system regulation. The widespread distribution and varied expression of AM in the body underscore its versatility and importance in health and disease functioning.

Despite the various research carried out and due to its versatility, the role of this molecule is not yet well defined. In recent years, some studies have investigated the role of ADM as a prognostic and predictive marker of negative outcomes in various diseases, such as clinical worsening and death. The aim of this work is to deepen the role of ADM as a prognostic marker with respect to the variable of death. Finally, we propose the results of a literature review based on the evaluation of experimental studies that investigated the role of ADM as a prognostic factor with respect to death. This review aims to analyze the potential of the molecule in the prediction of unfortunate clinical events and also analyze the physiological perspectives and therapeutic applications.

## 2. Materials and Methods

A narrative review of the literature was performed through the scientific search engines PubMed and Scopus. The following keywords were analyzed: “Adrenomedullin AND (death OR agony)”. Works published in English in the last 10 years were included in the search. Papers that were not strictly related to the prognostic function of the marker and the risk of negative clinical outcome were excluded from the search. The titles of the papers were analyzed first and then the abstracts were screened. The papers that met the inclusion criteria were analyzed with full-paper reading.

## 3. Results

**Figure 1:**
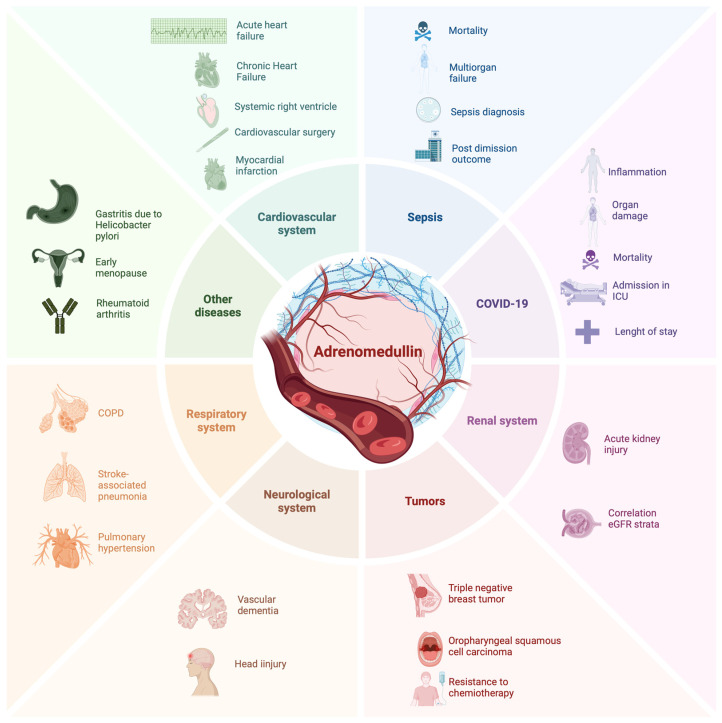
Predictive role of ADM.

This review yielded a total of over 700 works. The search yielded a total of 43 works that analyzed variables such as risk of death, predictability of adverse clinical events, and clinical worsening of the disease (Figure 1). This review did not yield any works that specifically examined agony as a comparison variable.

### 3.1. Role of ADM in Cardiovascular Mortality

Adrenomedullin has been proposed as a predictive marker of mortality in several types of cardiovascular disease. Among these, it is known that adults with systemic right ventricle are at high risk of mortality and congestion [2]. Since several studies highlighted that bio-ADM concentration is at the same level in the left ventricle and in the right ventricle, it has been hypothesized that in patients affected or those with an increased risk of mortality there may be an increase in circulating levels of this marker [11]. Through a trial on 85 patients, some authors have demonstrated that bio-ADM can improve risk prediction compared to pro-BNP alone and, therefore, it could be a marker of congestion and an independent predictor of death [2]. In the context of acute heart failure, Beer et al. have recently shown that high levels of pro-ADM can significantly predict the worsening of the disease and also the risk of death [4]. In the context of chronic heart failure, this molecule is associated with an increase in the risk of death and hospitalization [5,6]. The cited studies demonstrate the potential that ADM could offer in the prognostic evaluation of heart failure, especially with respect to the mortality risk of this disease. However, in cardiovascular pathology, ADM has demonstrated a predictive potential for other types of diseases. In another study, in a group of 67 patients affected by heart failure and pulmonary hypertension, before and after heart transplantation, it was evaluated that patients with high pre-operative levels of ADM have an increased mortality index compared to patients who showed lower levels [7]. This suggests that ADM may be a marker of pressure/volume overload in heart failure and a long-term prognostic marker after transplantation [7]. Another fundamental prognostic aspect in patients with cardiac comorbidities is represented by hospital discharge and, therefore, by the risk of death after hospitalization. Identification of patients with out-of-hospital cardiac arrest is also very important, because these patients develop various complications including inflammation, myocardial dysfunction with failure, and hypoxic brain damage. Among other possible applications, we highlight the possibility of using the MR-ADM marker in risk stratification in patients with out-of-hospital cardiac arrest. Even in ST-segment elevation myocardial infarction, the levels of this marker may act as an independent predictor of adverse cardiac events in the 90-day follow-up. This consideration arises from evidence in a study of significantly higher levels in patients who developed cardiac events 30 and 90 days after the primary infarction [12]. The results were confirmed by another study that evaluated the 2-year risk of acute cardiovascular events in patients with myocardial infarction [13,14]. These data highlight the diagnostic prospects of the marker in predicting long-term adverse events. This predictive potential of ventricular dysfunction is also present in patients who must undergo major surgery. For example, the predictive potential with respect to coronary artery bypass grafting was evaluated as demonstrated by monitoring the levels in a cohort of 93 subjects [8]. Also, in neonatal cardiac surgery, the predictive potential of mortality and short-term fatal complications was considered, as well as in aortic surgery by evaluating the risk of in-hospital death and cardiogenic shock [10]. The data suggest that ADM is, therefore, useful in the prognostic stratification of cardiac surgery risk in adult and neonatal cardiac pathologies.

Other possible applications concern the possibility of predicting the risk of arrhythmic death in patients with heart failure. This was demonstrated in a prospective observational study that evaluated 160 patients with heart failure and 30 control patients without heart failure, in order to estimate the risk of arrhythmic death based on the levels of the marker [9]. The study, which examined a total of 48 arrhythmic events, demonstrated significantly higher levels of MR-proADM in patients with ischemic and dilated cardiomyopathy compared to the control group. Furthermore, in patients with ischemic cardiomyopathy, the levels were associated with an increased risk of arrhythmic death [9]. The data are relevant as arrhythmic events represent one of the most frequent causes of mortality, often with unpredictable timing and methods in chronic heart disease patients. Therefore markers that can predict the risk of such adverse cardiac events are necessary in order to enhance surveillance and follow-up of these patients. Among other applications, we highlight how the combined use of MR-proADM and NT-proBNP can allow a more precise placement of patients with heart failure in remote patient management rather than in hospitalization [15,16].

Odermatt et al. evaluated, through a post hoc analysis, a correlation of proADM with 10-year all-cause mortality in a cohort of 134 patients with respiratory tract infections. In particular, median proADM levels were significantly higher in the non-survivor group than in the survivor group (0.5, IQR 0.4–1.3; vs. 0.2, IQR 0.1–0.5; *p* = 0.02) [13]. This proves the role of the marker as a prognostic index also in the prediction of long-term mortality.

### 3.2. Correlation with Mortality in COVID-19

Stratification of organ damage is a fundamental parameter for estimating the risk of mortality in COVID-19 patients for the purpose of the intensity of therapeutic measures to be adopted.

This review demonstrated that high levels of ADM were measured in patients with COVID-19 pneumonia who were in sub-intensive care or who died, demonstrating the potential of the marker as an indicator of levels of inflammation or organ damage [17,18]. In COVID-19 patients, a correlation with a significant increase in bio-ADM was also demonstrated in patients with critical acute kidney injury [19]. Data from another study revealed significantly higher levels in non-survivors compared to survivors, establishing that a value of mid-regional pro-adrenomedullin (MR-proADM) > 1.5 nmol/L is an independent mortality factor. These results have been substantially confirmed also in other studies comparing marker of survivors against non-survivors [20,21,22,23,24]. The data indicate that ADM represents a predictive mortality in COVID-19 disease.

Sozio et al. evaluated this potential as a prognostic factor in COVID patients, especially in combination with age and CRP levels or with SOFA score levels in order to identify low-risk patients compared to higher-risk patients through a retrospective observational analysis on 1861 patients [21]. These results have also been confirmed in other studies that examined the prognostic value in 116 hospitalized patients through retrospective data analysis, compared to the mortality index in combination with copeptin. Another study with 135 patients also evaluated this correlation with respect to the length of stay, death, and admission to intensive care unit indices [23]. This confirms the potential of ADM as a risk stratifier [23].

### 3.3. Correlation with Clinical Worsening and Death in Sepsis

In the context of sepsis, with experimental approaches similar to what has already occurred in the context of COVID-19 disease, the studies carried out have compared the levels of ADM in survivor and non-survivor groups admitted to the ICU, evaluating the association with different variables such as length of hospital stay, short-term mortality, and outcome after hospitalization [25,26,27,28].

Cander et al. demonstrated a significant correlation of the marker in the group of patients with sepsis compared to the control group as demonstrated by an increased length of hospital stay and mortality [25]. Another study considered 104 patients with severe sepsis, of which 39 died. Of these, the plasma levels of MR-proADM were measured, revealing an increase in the median values in the non-survivor group compared to the survivor one. The authors identified a cut-off of 1425 nmol/L for the diagnosis of sepsis. A value equal to or greater than 5626 nmol/L has also been associated with a high risk of mortality [26]. The data demonstrate how in clinical practice, particularly in patients suffering from organ dysfunction, ADM can be not only a prognostic marker of mortality but also a diagnostic marker. Using a similar approach, Charles et al. demonstrated, in a prospective cohort study carried out on 173 patients with sepsis in the ICU, that MR-ProADM values at the time of admission to the ward represent the best predictor of short-term outcome [27]. This result was achieved by measuring the levels of the marker from day 1 to day 5, with statistically significant differences in the non-survivor group compared to the survivor group [27]. In another similar study, the levels of the markers measured in a cohort of 53 patients at 7 and 28 days from admission were evaluated in a cohort of 53 patients with septic shock [28]. The results demonstrated that the levels were higher in the non-survivors compared to the control group [28]. Therefore, the literature show similar analysis strategies among various researches, through a comparison of marker levels in patients with sepsis in the ICU with analysis of various variables for diagnostic and predictive purposes.

### 3.4. Correlation with Worsening Renal Function

**Figure 2:**
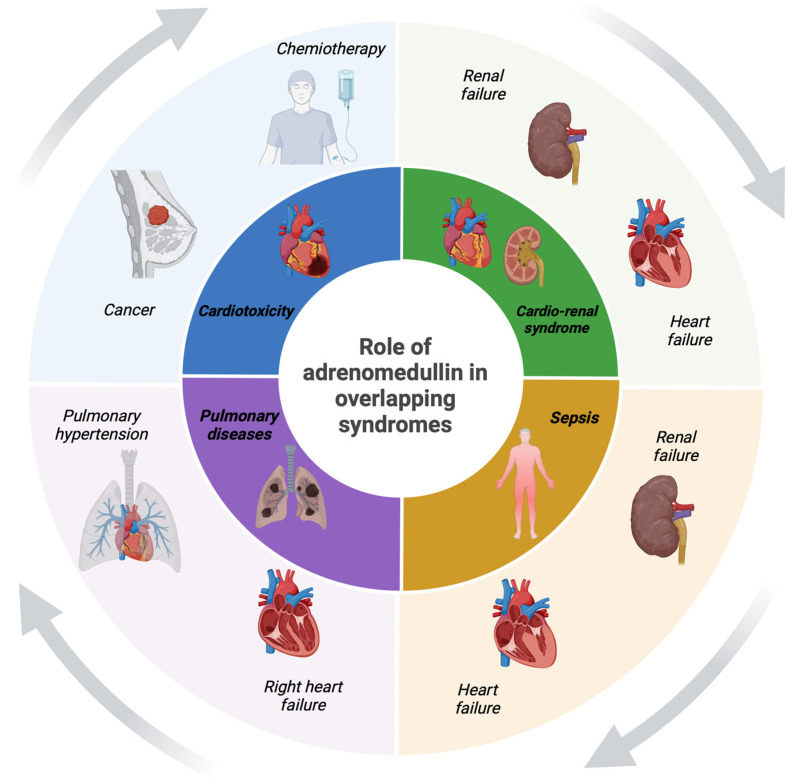
The role of ADM in overlapping diseases.

Several studies have evaluated how ADM is a marker related to renal damage [18,29]. Leininger et al. measured marker levels in 153 patients affected by COVID-19 admitted to the emergency department, taking as endpoints the presence of acute kidney injury (AKI) [18]. The results showed significantly high marker levels in patients affected by AKI compared to other COVID-19 patients. Furthermore, according to ROC analyses, ADM demonstrated the largest AUC (0.81) regarding the detection of AKI [18]. Mörtberg et al. recently demonstrated how ADM can increase in patients affected by acute renal failure [29]. The authors measured plasma levels in 1370 patients affected by acute coronary syndrome by spectrometry. The results showed an interaction of ADM values with eGFR strata (*p* = 0.010) and increased risk of cardiovascular events in patients with eGFR ≥ 60 mL/min/1.73 m^2^ [29]. The data suggest the usefulness of the marker in the risk stratification of renal failure and in the evaluation of overlapping syndromes such as renal failure and heart failure (Figure 2).

The correlation between MR-proADM and eGFR in chronic kidney disease patients underscores the peptide’s importance in renal health.

Confirming this, a proteomic approach allowed, among a panel of 175 different markers, the identification of six markers, including adrenomedullin, able to predict long-term outcomes in patients with renal failure and myocardial infarction [30]. The analysis included a total of 1098 patients affected by myocardial infarction with an eGFR of 85 mL min^−1^/1.73 m^2^. The analyses demonstrated that ADM was among the strongest markers associated with CKD (chronic kidney disease) [30].

Kakareko et al. examined 77 patients admitted to the ICU, measuring their levels in the first 24 h after admission. The results showed that MR-proADM was not useful in predicting incident AKI in the general ICU population [31].

Another study demonstrated how ADM plays a protective role in the kidney. In particular, its receptor AM-RAMP2 is able to suppress ER stress-induced tubule cell death, playing a protective role on the kidney [32]. In this field, Artunc et al. measured plasma levels of MR-pro-ADM in 239 outpatients undergoing hemodialysis, evaluating the association between mortality and clinical factors. The study showed that in all patients analyzed, the levels were high. Furthermore, the concentration correlated with the dialysis time and with the concentration of β2-microglobulin, which was also predictive of mortality [33,34].

Evidence suggests that ADM significantly impacts tubular function and water balance within the kidneys. AM’s diuretic action facilitates the excretion of excess water, contributing to the regulation of fluid balance and preventing conditions such as edema. This diuretic effect is complemented by AM’s role in maintaining sodium homeostasis, which is crucial for overall fluid balance and blood pressure regulation. By promoting both water and sodium excretion, AM helps to ensure the kidneys operate efficiently, maintaining homeostasis and preventing fluid overload.

### 3.5. Correlation with Worsening of Neoplastic Diseases and Resistance to Chemotherapy

Various studies have demonstrated a marker dysregulation in various types of tumors such as osteosarcomas, pancreatic adenocarcinomas, prostate cancer, and gastric adenocarcinoma [35,36,37,38].

In particular, in the latter, an overexpression of the marker is associated with a worse prognosis. For this reason, it has been proposed that the marker could also be a therapeutic target capable of stimulating vascular neoangiogenesis in neoplastic diseases. However, the literature shows that the expression of the marker in various tumors is not always the same and does not always have the same meaning. For example, a study has shown, through in vitro analysis, the properties of the marker in the inhibition of tumor metastases in tissues of patients affected by triple-negative breast cancer [39]. This result was achieved by measuring the expression levels of ADM in patients affected by triple-negative breast cancer by Western blotting. In this tumor, the tissue samples affected by breast cancer presented low levels of this marker and the levels were even lower in the case of metastases. In particular, the authors demonstrated how low levels of ADM in triple-negative breast cancer are associated with an overall poor prognosis [39]. These characteristics suggest that ADM is a biomarker for the prognosis of triple-negative breast cancer (TNBC) and that they may be a candidate therapeutic target for anti-metastasis [39].

In oropharyngeal squamous cell carcinoma, ADM expression has been associated with bad prognosis. Maia et al. evaluated ADM levels in paraffin-embedded tissues from 84 patients with oral and oropharyngeal carcinoma by immunohistochemical investigations and evaluated a predictive correlation with the progression and prognosis of these tumors. In particular, it was seen that ADM is associated with a higher risk of metastasis [40].

The marker also seems to be associated with resistance to chemotherapy. In renal cell carcinoma, ADM is able to promote resistance to sunitinib, through the reduction of FDX1 expression levels, thus inhibiting cuproptosis [41]. In another study, Yoshizawa demonstrated that ADM is able to protect against cardiac damage induced by doxorubicin, a drug used in various tumors including gastric, lung, and lymphoma. In particular, exogenous administration of ADM in rats has shown an improvement in the survival ratio with a protective effect [42].

### 3.6. Correlation with Clinical Worsening of Other Diseases

Over the last 10 years, other correlations between ADM marker levels and other pathologies that are associated with cardiovascular damage or alteration of vascular integrity have been carried out.

It has been shown that early menopause shows an increase in circulating levels of proteins associated with cardiovascular diseases, capable of predicting the risk of adverse cardiovascular events. This, therefore, suggests a correlation of this disorder with cardiovascular diseases. Among these proteins, a correlation of ADM with early menopause has been evaluated by enrolling a cohort of over 1500 menopausal patients and analyzing the levels of the marker in early menopause [43].

Other authors have demonstrated in a pool of about 400 patients how ADM is associated with an increase in the duration of rheumatoid arthritis. In this study, this proves that arthritis is a pathology that also causes cardiovascular damage associated with an increase in the marker levels [44].

In the neurological field, a recent experimental study carried out on an animal model evaluated rats affected by head trauma, treated with ADM compared to a control group of rats with the same pathology but not treated. The experimental work demonstrated neuroprotective effects of this molecule, considering a lowering of GSH levels in the treated rats [45]. Thanks to its angiogenic role, the therapeutic potential of this molecule in vascular dementia was evaluated through the creation of an experimental model on rats. Here, they demonstrated how, in hypoxic conditions, ADM promotes the differentiation of oligodendrocytes, exerting a neuroprotective role and slowing down dementia [46]. These properties were also evaluated in another experimental animal study, which proved an in vitro neuroprotective effect from toxic agents, such as doxorubicin, in rat neurons [47].

In the field of predicting the clinical course of respiratory diseases, a study carried out by Zuur-Telgen et al. evaluated the marker as a predictor of mortality in patients affected by COPD. In particular, they examined over 262 patients affected by COPD and hospitalized for an episode of acute exacerbation [48]. The study evaluated how this marker could be even more promising than other already known markers such as fibrinogen [48]. Other studies have evaluated the potential to predict the risk of stroke-associated pneumonia and death from pulmonary hypertension [49].

This molecule is associated with inflammatory diseases and can also be activated by pathogenic agents such as microorganisms. For example, the pathogen Helicobacter pylori, a known agent responsible for gastritis, can determine a stimulation in the production of ADM correlating with the severity of the degree of gastritis [50]. Therefore, ADM shows, thanks to its ubiquity, a notable diagnostic and prognostic potential in a large number of diseases.

## 4. Discussion

### 4.1. Cardiovascular Activity

Adrenomedullin (ADM) plays a significant role in the regulation of vascular tone and blood pressure through its vasodilatory effects. ADM is produced and secreted by several tissues, including the heart, lungs, and kidneys, and it acts as an autocrine or a paracrine hormone [1,2,3,4]. It is particularly effective in the vascular wall, where it contributes to the maintenance of blood pressure homeostasis. Its mechanism of action involves the relaxation of vascular smooth muscle cells, leading to vasodilation and subsequently a decrease in blood pressure. This effect is a critical component of the body’s cardiovascular regulation, highlighting the importance of ADM in maintaining circulatory stability [1,2,3,4].

Beyond its role in vasodilation, adrenomedullin has demonstrated significant cardioprotective effects, particularly in the context of heart disease. Studies have shown that ADM can reduce several pathological mechanisms for its cardioprotective action such as oxidative stress and inhibit endothelial cell apoptosis [51]. Additionally, ADM is known to be produced in cardiac fibroblasts, which suggests that it exerts its myocardial effects directly within the heart. This peptide hormone thereby aids in protecting the heart from various forms of damage, including that induced by ischemia-reperfusion injury [52]. The ability of ADM to mitigate the detrimental effects associated with heart disease underscores its potential as a therapeutic target. It promotes the formation of new blood vessels and supports the structural integrity of existing ones, a process that is essential for tissue repair and regeneration [51]. This angiogenic function is primarily mediated through the activation of signaling pathways that stimulate endothelial cell proliferation and migration. ADM’s involvement in angiogenesis not only facilitates normal developmental processes but also plays a crucial role in the body’s response to injury, by ensuring an adequate blood supply to damaged tissues. Moreover, its ability to regulate vascular integrity helps to prevent excessive vascular permeability, further contributing to its protective effects in the cardiovascular system [14].

In healthy subjects, circulating levels of the ADM marker are low. It is likely that there are a large number of diseases related to vascular damage, in which there is a strong release of ADM. In the context of acute heart failure, some patients may present a progression of the disease towards cardiogenic shock, which is characterized for reduced systemic perfusion due to cardiac pump failure with signs such as possible hypotension, anuria, cold extremities, and altered mental status. The literature shows that ADM is a marker that increases in these circumstances, probably with the aim of preventing tissue volume overload [6,7,8,9,10,11,12,13,14,15,16]. Furthermore, ADM levels increase cardiac output, inducing hypotension and vasodilation, with a decrease in cardiac pre- and afterload.

This literature review, therefore, highlights how ADM has numerous protective properties related to the improvement and preservation of vascular integrity, reduction of vascular permeability, and organ damage. Thanks to its properties associated with vascular integrity, it is equally reasonable to believe that ADM levels are elevated in all pathologies related to vascular damage, hypoxia and cellular ischemia, and congestion, thus constituting an important indicator of the risk of cardiovascular mortality or high-risk of adverse clinical events. These properties suggest that, being a ubiquitous marker and since in the majority of deaths there is a primary involvement of the heart in the chain of events, there is a significant increase in its levels before death. These properties allow for predictability, which is essential in various clinical contexts, including risk stratification in cardiovascular surgery or monitoring the therapeutic success and patient prognosis in acute and chronic heart failure or even in the prediction of adverse cardiac events in patients with recent myocardial infarction [10,11,12,13,14,53].

Given its functions, it is reasonable to predict that ADM will have a preponderant role especially in therapy thanks to the use of targeted molecular antibodies, which can exploit the vasodilatory properties of the molecule. These functions can be used to personalize diuretic treatment, in patients with heart failure, or those with significant cardiac comorbidities.

### 4.2. The Role in Death from Sepsis

Sepsis is an organ dysfunction characterized by a dysregulation between the host response and the infection leading to a very high mortality rate (50–60%) [54]. None of the characteristic signs of sepsis can be considered pathognomonic. For this reason, there is an important need to identify economic and effective biomarkers capable of making the diagnosis and allowing early identification. In fact, according to the guidelines, the treatment of sepsis must be personalized, and in an emergency context, it is much easier to rely on a measurable marker with a blood sample to calculate a score that includes a lot of data. In fact, nowadays the most used scale is the SOFA score, which is used to evaluate organ damage and to predict mortality in patients with sepsis or septic shock [55].

In the review carried out, MR-proADM was proven to be an excellent alternative to the SOFA score, especially since it allows for triage stratification. Studies have shown that MR-proADM serves as a prognostic marker in sepsis, with its levels reflecting the severity of the disease and the degree of organ damage [25,26]. Furthermore, the combination of MR-proADM with other biomarkers such as procalcitonin (PCT) has been explored, revealing enhanced utility in diagnosing and prognosticating sepsis [25,26]. This research underscores the multifaceted role of ADM, not only as a therapeutic target but also as critical biomarker that aids in the early assessment and prediction of mortality in septic patients. This review also demonstrates how the diagnosis is often the result of the combination with other markers (Table 1).

### 4.3. The Role in Identifying Severe Forms of COVID-19

Since 2021, a series of papers have been published in which the roles of the ADM marker and its surrogates as a prognosis indicator also in COVID-19 disease were investigated [17,18,19,20,21,22,23,24]. The complex pathological process determined by COVID-19 is promoted by pulmonary endothelial cells that induce a pro-coagulative state, with endothelitis and tissue edema. ADM could play a key role in COVID-19-associated endothelitis [23]. In addition, ADM and more specifically its more stable surrogate MR-proADM, can be evaluated as a biomarker of severity in COVID-19 pneumonia. The marker also shows properties of inflammatory markers with short-term predictive potential of death, acute respiratory distress syndrome, multiorgan dysfunction, and progression to severe systemic disease [17,18,19,20,21,22,23,24] Therefore, this marker could be valuable in evaluating early diagnostic intervention regardless of the causative pathogen. In addition, some studies have suggested evaluating the PaO_2_/FiO_2_ ratio regarding oxygen supplementation before anesthesiologist evaluation because it is very difficult to measure this ratio [17,18,19,20,21,22,23,24]. In this context, MR-proADM plays an essential prognostic role, considering its role in the regulation of vascular permeability, inflammatory mediators, and microcirculation. Therefore, the marker could play a crucial role in monitoring the progression of COVID-19 disease, contributing to the correct triage of the COVID-19 patient by anticipating intensive treatments regardless of the responsible pathogen such as fungi, bacteria, and viruses. The marker could, therefore, predict the clinical outcome and therapeutic efficacy, allowing an early identification of clinical worsening. The literature suggests that this marker offers a higher accuracy than that offered by CRP and PCT in identifying the severity of the disease and the response to treatment, thus allowing identification of the multiple complications related to endothelialitis induced by SARS-CoV-2 virus including thromboembolism and acute respiratory distress [17,18,19,20,21,22,23,24].

### 4.4. Physiology of ADM in the Prognosis of Renal Diseases

ADM plays a crucial role in regulating renal blood flow and diuresis, embodying a series of renal vasodilatory, natriuretic, and diuretic actions that significantly impact kidney function. By increasing the glomerular filtration rate and renal blood flow, AM contributes to the efficient removal of waste products from the bloodstream and into the urine [56]. Intrarenal arterial infusion of ADM has been demonstrated to increase renal blood flow or glomerular filtration rate in animal studies, such as those conducted on dogs, leading to diuresis and natriuresis [56]. These findings underscore the importance of AM in the intricate regulation of kidney processes and highlight its potential therapeutic applications in conditions affecting renal blood circulation.

The protective effects of adrenomedullin in both acute kidney injury (AKI) and chronic kidney disease (CKD) are well documented, underscoring its significant renoprotective properties. Elevated levels of adrenomedullin have been suggested to play a compensatory role in CKD, helping to mitigate the progression of kidney damage [12]. Adrenomedullin significantly impacts electrolyte balance and blood volume, playing a pivotal role in the regulation of fluid and electrolyte status, including the inhibition of aldosterone secretion, which is crucial for maintaining osmolar balance and preventing excessive fluid retention [18]. Furthermore, the peptide’s natriuretic and diuretic properties reflect its ability to adjust body fluid levels and electrolyte balance, ensuring optimal physiological conditions [56].

It is known in the literature that renal function impairment represents an important risk factor for cardiovascular events. Patients with chronic renal failure are those subjects who show worse clinical outcomes compared to patients with preserved function. Impaired renal function is also associated with both arterial and venous thrombotic events, risk of bleeding, and alteration of the coagulation cascade. Endothelial function is also impaired in chronic kidney disease and may contribute to a proinflammatory, procoagulant state. In the studies analyzed, ADM showed a correlation of its concentration with an increase proportional to the decrease in eGFR [29,30,31,32,33,34]. Furthermore, the literature has demonstrated an association between the timing of dialysis, accumulation of β2-microglobulin concentrations, and a protective cellular function of ER stress-induced tubule cell death in the kidney [29,30,31,32,33,34]. Therefore, these data suggest that ADM plays a prognostic and a predictive role also in the setting of renal failure, although further studies are needed to integrate the marker in the context of a more precise prediction of the risk of acute and chronic kidney damage with use in clinical practice. In this context, ADM could also play a potential role as a prognostic marker for diuretic resistance. This is an important issue, especially for prognosis and choice of treatment strategy for heart failure, with or without renal failure.

### 4.5. Function of ADM in the Prognosis of Neoplastic Diseases

Adrenomedullin (AM) plays a pivotal role in cancer progression, acting not only as a regulatory peptide but also as a potential biomarker for tumor development and metastasis [38,39]. Its production and secretion are not limited to tumor cells; a broad array of stromal cells including macrophages, mast cells, endothelial cells, and vascular smooth muscle cells are also involved in its synthesis. This broad spectrum of cellular sources underlines the complexity of AM’s involvement in tumor biology. Moreover, the peptide’s regulatory functions in tumor progression and metastasis have been increasingly recognized in recent studies [35,36,37,38], highlighting its dual role in both promoting and potentially inhibiting cancer development. This intricate involvement of AM in cancer makes it a promising target for biomarker research and therapeutic strategies, as understanding its precise role could lead to novel approaches in cancer diagnosis and treatment.

The literature shows that the production of this marker represents a good investment for the tumor, being able to act as a growth factor, to increase tumor cell motility and metastases but also inducing neoangiogenesis [57]. Furthermore, the literature suggests that its expression could increase rapidly due to its activation by hypoxia and transcription factors such as HIF-1. Certainly, a large number of studies will be needed in order to clarify the complex network of molecular interactions associated with ADM in the tumor and the possibility of suppressing it with new molecular targets.

### 4.6. Correlation with Prognosis of Other Diseases

ADM has demonstrated an association with other anatomical areas and pathologies, although most studies are focused on the cardiovascular area and on the infectious risk in sepsis [43,44,45,46,47,48,49,50]. The ubiquity of this molecule and especially how its physiology closely correlates with vascular damage suggests, in accordance with the literature, that ADM may be a prognostic marker involved in the physiopathology of many diseases and in any anatomical system, significantly varying its concentrations as a function of variables related to vascular damage as well as inflammation, angiogenesis, and organ damage. These functions, therefore, suggest significant clinical implications in the context of risk stratification of mortality and adverse clinical outcomes.

### 4.7. Clinical Trials Devoted to Treatment with ADM

Ongoing clinical trials are exploring the potential applications of adrenomedullin in various medical conditions. For instance, a current phase II study aims to evaluate the use of ADM as a treatment option for ischemic stroke and sepsis, conditions characterized by extreme immune reactions and endothelial dysfunction [58]. Another notable phase II trial is investigating the efficacy of AM in patients with CADASIL, a hereditary small vessel disease that leads to recurrent strokes and dementia [59]. This trial seeks to leverage the vasoactive properties of AM to alleviate the symptoms of this debilitating condition.

The findings from an investigator-initiated phase 2 trial have shown promising results for the treatment with intravenous (IV) ADM. This study demonstrated that IV AM could significantly improve patient outcomes by enhancing endothelial function and reducing systemic inflammation [60]. Additionally, in vitro and preclinical in vivo data support these findings, revealing that AM administration exerts anti-inflammatory, antimicrobial, and protective effects on the endothelial barrier [61]. These effects are particularly relevant in conditions where endothelial integrity is compromised, such as in sepsis and acute ischemic stroke. The anti-inflammatory properties of AM were further corroborated by a clinical trial that focused on its use for inflammatory bowel disease, where it was found to suppress inflammatory cytokine production in the intestinal mucosa and improve vascular and lymphatic regeneration and function [62].

### 4.8. Therapeutic Application of ADM Antibodies

ADM antibodies are emerging as a promising therapeutic approach in the treatment of cardiovascular disorders. For instance, the long-acting derivatives of ADM have been developed to address the limitations of short-term treatments in cardiovascular diseases [63]. This innovative approach not only improves the effectiveness of the treatment but also enhances patient compliance by reducing the frequency of administration. Moreover, these antibodies can potentially inhibit vascular inflammation and improve hypertension and vascular remodeling. By targeting ADM, these therapies offer a novel mechanism to manage and treat various cardiovascular conditions more effectively.

The application of ADM antibodies in managing septic shock has also shown significant potential. Preclinical studies have demonstrated that antibodies binding to the N-terminus of ADM lead to an overall increase in circulating ADM levels, which can benefit patients with septic shock [64]. Furthermore, the use of non-neutralizing ADM-binding antibodies, such as adrecizumab, has yielded promising results in animal models. A study reported improved haemodynamic parameters and reduced myocardial oxidative stress, which are critical in managing septic shock [65]. Additionally, preclinical work has highlighted the potential of these treatments to enhance vascular barrier function and reduce organ dysfunction in septic patients [66]. Therefore, ADM antibodies represent a promising therapeutic avenue for improving outcomes in septic shock management.

In the context of cancer therapy, anti-ADM antibodies offer a novel approach to combating tumor growth and angiogenesis. Studies indicate that ADM plays a significant role in promoting tumor growth and influencing tumor microenvironments [67]. Anti-ADM therapy, therefore, can be a promising strategy, particularly in the context of acquired resistance to traditional anti-angiogenic treatments [68]. By targeting the AM pathways, these antibodies can potentially inhibit tumor progression and enhance the efficacy of existing cancer therapies. Furthermore, focusing on the ADM-2 receptor complex can minimize off-target effects and improve treatment specificity [57]. This targeted approach could revolutionize cancer therapy, providing new hope for patients with resistant or aggressive tumor types.

Additionally, ADM antibodies have exhibited beneficial effects in rodent models of inflammatory bowel disease (IBD) and have been tested in clinical trials involving human patients [69]. Furthermore, ongoing trials aim to establish the safety and tolerability of adrecizumab, thereby strengthening its clinical viability. These studies collectively highlight the therapeutic potential of AM antibodies across various inflammatory conditions.

Looking ahead, the future perspectives and potential new indications for ADM antibodies are expansive.

### 4.9. Future Perspectives

Future ADM research perspectives aim to include the measurement of the marker in laboratory investigations in hospitals. This measurement is especially desirable in intensive care settings with patients suffering from sepsis or in any case in clinical contexts where there is a strong need for mortality risk stratification, such as in geriatric or surgical settings. The potential for new treatment and clinical trials involving ADM is immense, offering hope for conditions that have long eluded effective treatment. These possibilities could materialize thanks to new potential treatment strategies with anti-ADM antibodies in a multitude of areas, including the cardiovascular, infectious disease, or oncology fields.

## 5. Conclusions

The predictive value of adrenomedullin in determining the outcomes of both acute and chronic diseases has been extensively documented, providing invaluable insights for clinical decision-making. In a large number of pathologies, adrenomedullin levels not only correlate with the severity of the disease but also with mortality rates, illustrating the biomarker’s comprehensive predictive capability across various disease states [5]. These findings underscore the versatility of adrenomedullin as a predictive biomarker for a wide range of medical conditions, highlighting its potential to guide therapeutic strategies and to improve patient outcomes. When compared to other biomarkers in mortality predictions, adrenomedullin exhibits a comparable, or even superior, predictive value.

It is plausible to assume that all the pathologies examined are likely associated with severe endothelial damage with vascular imbalance and attempt to regulate and restore homeostasis by producing ADM. These results demonstrate how ADM can constitute a marker with varied clinical prognostic and predictive applications of the risk of death or of adverse clinical outcomes. With regards to agony, this review did not find works associated with this specific variable. Future research directions are, therefore, aimed to the possibility of extending the prediction of the risk of death also in diseases other than those investigated up to now, with the possibility of expanding the clinical and therapeutic applications.

## Figures and Tables

**Table 1:** Prognostic role of ADM in combination with other markers.

Associated Marker	Prognostic Value
NT-proBNP	Hospitalization in heart failure
CRP	Severity of COVID-19
Procalcitonin	Clinical evolution of sepsis
